# Synthetic, Mesomorphic, and DFT Investigations of New Nematogenic Polar Naphthyl Benzoate Ester Derivatives

**DOI:** 10.3390/ma14102587

**Published:** 2021-05-16

**Authors:** Salma A. Al-Zahrani, Hoda A. Ahmed, Mohamed A. El-atawy, Khulood A. Abu Al-Ola, Alaa Z. Omar

**Affiliations:** 1 Department of Chemistry, College of Sciences, University of Ha’il, Ha’il 2440, Saudi Arabia; s.alzahrane@uoh.edu.sa; 2 Department of Chemistry, Faculty of Science, Cairo University, Cairo 12613, Egypt; 3 Chemistry Department, Faculty of Science, Taibah University, Yanbu 46423, Saudi Arabia; 4 Chemistry Department, Faculty of Science, Alexandria University, P.O. 426 Ibrahemia, Alexandria 21321, Egypt; alaazaki@alexu.edu.eg; 5 Department of Chemistry, College of Science, Taibah University, Al-Madinah Al-Munawarah 30002, Saudi Arabia; kabualola@taibahu.edu.sa

**Keywords:** fused ring, azo/ester, mesomorphic properties, liquid crystals materials, optimized structures, DFT, thermal parameters

## Abstract

Four new non-symmetrical derivatives based on central naphthalene moiety, 4-((4–(alkoxy)phenyl) diazenyl)naphthalen–1–yl 4–substitutedbenzoate (**I_n/x_**), were prepared, and their properties were investigated experimentally and theoretically. The synthesized materials bear two wing groups: an alkoxy chain of differing proportionate length (n = 6 and 16 carbons) and one terminal attached to a polar group, X. Their molecular structures were elucidated via elemental analyses and FT-IR and NMR spectroscopy. Differential scanning calorimetry (DSC) and polarized optical microscopy (POM) were carried out to evaluate their mesomorphic properties. The results of the experimental investigations revealed that all the synthesized analogues possess only an enantiotropic nematic (N) mesophase with a high thermal stability and broad range. Density functional theory (DFT) calculations were in accordance with the experimental investigations and revealed that all prepared materials are to be linear and planar. Moreover, the rigidity of the molecule increased when an extra fused ring was inserted into the center of the structural shape, so its thermal and geometrical parameters were affected. Energy gap predictions confirmed that the **I_16/c_** derivative is more reactive than other compounds.

## 1. Introduction

Calamitic liquid crystals (LCs) are widely used in LC displays and optical systems because of the suitability of their anisotropic properties [1,2,3]. However, their thermal and optical properties, though, can be adjusted by modifying the molecular geometries of the mesogenic compounds. Therefore, to develop a new LC material, the correlation between the geometry of its mesogenic part and its mesomorphic properties should be understood. Several calamitic azo/ester LC derivatives have been investigated and evaluated based on their optical properties [4,5]. From these geometrical investigations, one can determine the ester orientation within the rigid portion, the location of azo/ester linkages, lateral groups, and the terminal flexible–chain length [6,7,8,9,10,11,12,13]. A rigid shape creates azobenzene molecules, which are essential for exhibiting mesomorphic phenomena [14,15]. Moreover, their properties may lead to molecular mobility in terms of response to light or heat, thus offering many opportunities for photonic application [16,17,18,19,20,21].

It was found that the insertion of a lateral group on the aromatic ring of the mesogenic part influenced the mesophasic transition temperature range of the smectic phases [22,23,24] and increased spontaneous polarization [25]. The existence of mesophases near room temperature is very important for potential applications; consequently, lateral moieties are incorporated into the molecular architecture to lower the melting temperature [26]. On the other hand, polar terminal substituents and linking moieties were both important factors for the designed compounds, in terms of the formation, kind, thermal stability, and range of the observed mesophase [5,12,27,28,29,30,31,32,33,34,35,36,37,38,39,40].

Simulations of computational calculations for the molecular-shaped parameters offered interesting correlations between the experimental findings and theoretical predictions [41,42,43,44,45,46,47,48,49,50,51,52].

**Scheme 1:**
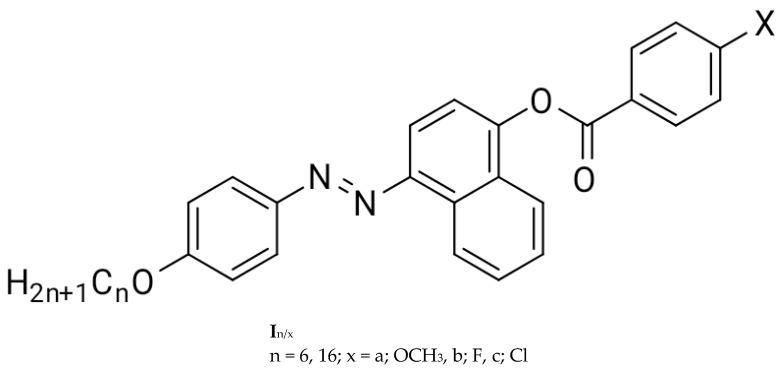
The synthetic compounds, **I_n/x._**

Recently, many homologous series have been documented based on naphthalene-core LCs [53,54,55,56,57]. In our previous study [45], a homologous series of (E)-4-(4-(hexyloxyphenyl diazenylnaphthalene-1-yl-4′-alkoxybenzoate was synthesized and its mesomorphic properties evaluated. The recently documented series were found to exhibit an enantiotropic nematic (N) mesophase. Moreover, our research lab has focused its attention on the computational investigations of newly, synthesized liquid crystalline materials to correlate their mesomorphic behavior with the theoretical calculations. The geometrical structure of LCs plays an essential role on the thermal stability and formation of their mesophases. Additionally, it has been found that the addition of extra fused ring in the mesogeic portion of the molecule results a new geometrical property and nematic mesophase predominant observed [45]. Further, the length of alkoxy terminal chains has essential role in the formation, stability, kind, and mesomorphic range of LC derivatives. In this investigation, we extended our studies to evaluate the effect of additional terminal polar substituents having a different polarities and sizes. The aim was to synthesize new derivatives based on a central naphthalene group (**I_n/x_**) bearing two terminals (the alkoxy proportion group and a terminal attached to a polar substituent (X), Scheme 1) to investigate their mesomorphic properties via experimental and theoretical approaches.

## 2. Experiment

### Synthesis

**Scheme 2:**
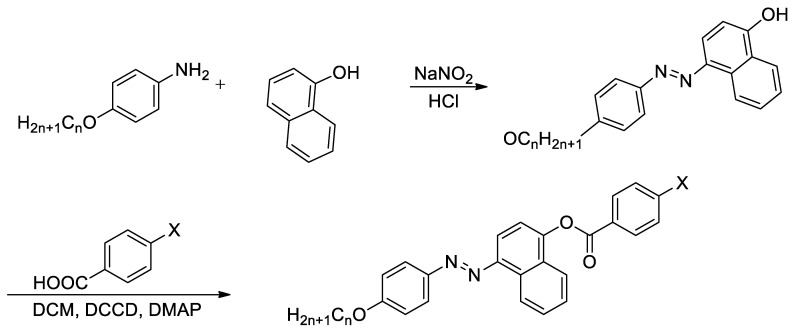
Synthesis of 4-((4-(alkoxy)phenyl)diazenyl)naphthalen-1-yl 4-substitutedbenzoate, **I_n/x._**

The liquid crystalline compounds **I_n/x_** were synthesized according to the following Scheme 2.

The synthetic and characterization details are described in the Supplementary Materials.

**4-((4-(hexyloxy)phenyl)diazenyl)naphthalen-1-yl 4-methoxybenzoate, I_6/a_**

**Figure 1:**
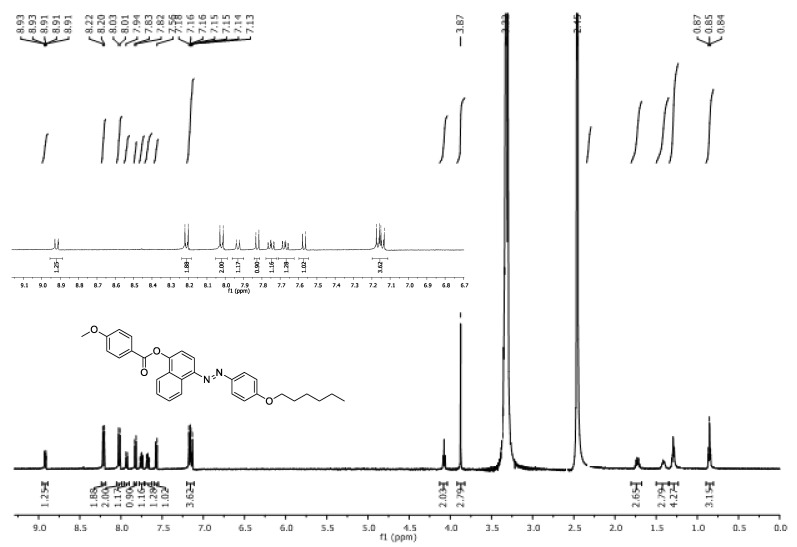
^1^H NMR of 4-((4-(hexyloxy)phenyl)diazenyl)naphthalen-1-yl 4-methoxybenzoate **I_6/a_** as a prototype.

Yield: 96.3%; mp 129.0 °C, FTIR (ύ, cm^−1^): 2960, 2830 (CH_2_ stretching), 1725 (C=O), 1602 (N=N), 1160 (C−O_Ester_), 1080 (C–O_Alkoxy_). ^1^H NMR, Figure 1, (500 MHz, (CD_3_)_2_SO) δ 8.93–8.91 (m, 1H, Naph–H), 8.21 (d, *J* = 8.9 Hz, 2H, Naph–H), 8.02 (d, *J* = 9.0 Hz, 2H, Naph–H), 7.93 (d, *J* = 7.8 Hz, 1H, Ar–H), 7.82 (d, *J* = 8.2 Hz, 1H, Ar–H), 7.75 (ddd, *J* = 8.4, 6.8, 1.2 Hz, 1H, Ar–H), 7.69–7.65 (m, 1H, Ar–H), 7.57 (d, J = 6.6 Hz, 1H, Ar–H), 7.19–7.13 (m, 4H, Ar–H), 4.10–4.05 (m, 2H, OCH_2_), 3.87 (s, 3H, OCH_3_), 1.78–1.65 (m, 2H, CH_2_), 1.47–1.35 (m, 2H, CH_2_), 1.34–1.23 (m, 4H, 2CH_2_), 0.85 (t, *J* = 7.1 Hz, 3H, CH_3_). Elemental Analysis calc. (found): C, 74.67 (74.65); H, 6.27 (6.24); N, 5.81 (5.80).

**4-((4-(hexadecyloxy)phenyl)diazenyl)naphthalen-1-yl 4-methoxybenzoate, I_16/a_**

Yield: 91.2%; mp 87.0 °C, FTIR (ύ, cm^−1^): 2935, 2845 (CH_2_ stretching), 1735 (C=O), 1590 (N=N), 1162 (C−O_Ester_), 1080 (C–O_Alkoxy_). ^1^H NMR (500 MHz, (CD_3_)_2_SO) δ 8.95–8.92 (m, 1H, Naph–H), 8.23 (d, *J* = 8.9 Hz, 2H, Naph–H), 8.00 (d, *J* = 9.0 Hz, 2H, Naph–H), 7.90 (m, 1H, Ar–H), 7.82 (d, *J* = 8.2 Hz, 1H, Ar–H), 7.75 (m, 1H, Ar–H), 7.69–7.65 (m, 1H, Ar–H), 7.57 (d, J = 6.6 Hz, 1H, Ar–H), 7.19–7.13 (m, 4H, Ar–H), 4.10 (t, *J* = 7.7 Hz, 2H), 3.85 (s, 3H), 1.77–1.65 (m, 6H), 1.60–1.54 (m, 4H), 1.49–1.33 (m, 4H), 1.26–1.15 (m, 6H), 1.11–0.94 (m, 8H), 0.77–0.67 (m, 3H). Elemental Analysis calc. (found): C, 77.14 (77.13); H, 8.09 (8.08); N, 4.50 (4.47).

**4-((4-(hexadecyloxy)phenyl)diazenyl)naphthalen-1-yl 4-chlorobenzoate, I_16/c_**

Yield: 92.7%; mp 107.0 °C, FTIR (ύ, cm^−1^): 2944, 2848 (CH_2_ stretching), 1725 (C = O), 1595 (N = N), 1162 (C−O_Ester_), 1080 (C–O_Alkoxy_). ^1^H NMR (500 MHz, (CD_3_)_2_SO) δ 8.90 (m, 1H, Naph–H), 8.23 (m, 2H, Naph–H), 8.00 (m, 2H, Naph–H), 7.90 (m, 1H, Ar–H), 7.85 (d, *J* = 8.1 Hz, 1H, Ar–H), 7.78(m, 1H, Ar–H), 7.68–7.63 (m, 1H, Ar–H), 7.57 (m, 1H, Ar–H), 7.18–7.10 (m, 4H, Ar–H), 4.12 (t, 2H), 1.77–1.53 (m, 6H), 1.49–1.32 (m, 4H), 1.29–1.17 (m, 4H), 1.09–0.97 (m, 8H), 0.91 (m, 6H), 0.74 (m, 3H). Elemental Analysis calc. (found): C, 74.68 (74.66); H, 7.55 (7.54); Cl, 5.65 (5.63); N, 4.47 (4.45).

**4-((4-(hexyloxy)phenyl)diazenyl)naphthalen-1-yl 4-fluorobenzoate I_6/b_**

Yield: 93.0%; mp 114.0 °C, FTIR (ύ, cm^−1^): 2950, 2846 (CH_2_ stretching), 1726 (C=O), 1592 (N=N), 1158 (C−O_Ester_), 1074 (C–O_Alkoxy_). Elemental Analysis calc. (found): C, 74.02 (74.02); H, 5.78 (5.75); F, 4.04 (4.03); N, 5.95 (5.94).

## 3. Results and Discussion

### 3.1. Mesomorphic and Optical Behaviour

**Figure 2:**
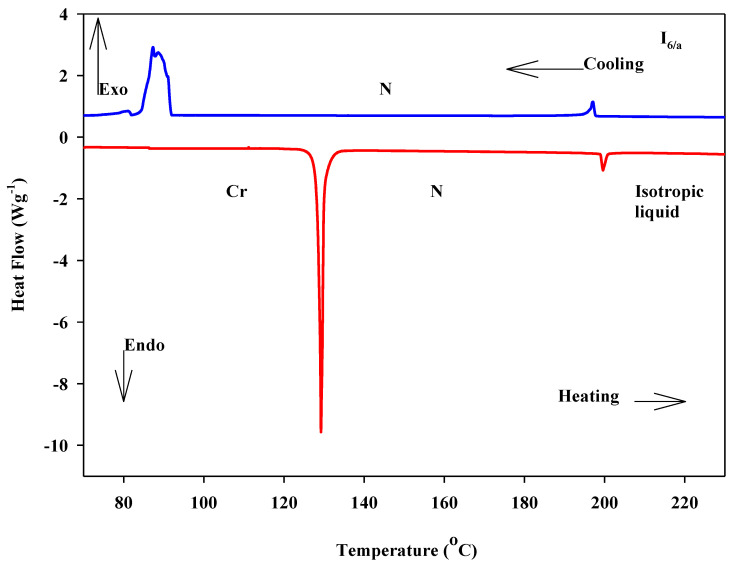
The second heating and cooling scan of a compound **I_6/a_** DSC thermogram at a heating rate of 10 °C min^−1^.

**Figure 3:**
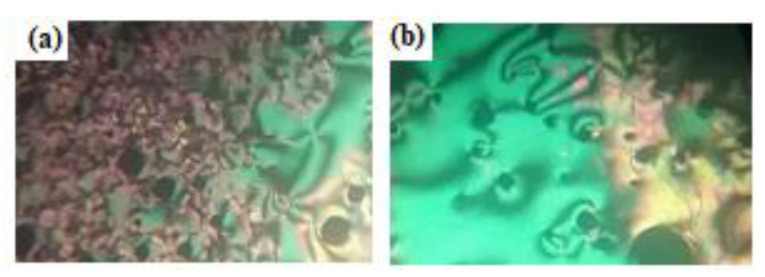
Nematic textures under POM on heating of the compound **I_16/c_** at (**a**) 130.0 °C and (**b**) 150.0 °C.

**Figure 4:**
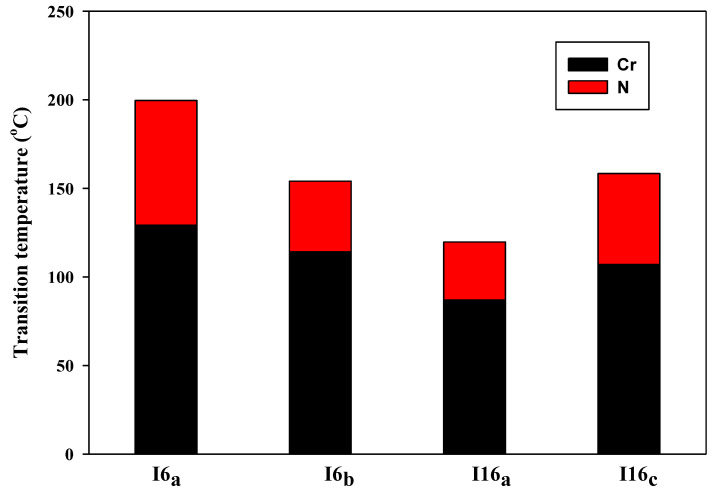
DSC transitions of the derivatives, **I_n/x_**.

The mesomorphic and optical characteristics of the present azo/ester derivatives, (**I_n/x_**) were investigated by differential scanning calorimetry (DSC) and polarized optical microscopy (POM). Figure 2 shows an example of the DSC thermograms of 4-((4-(alkoxy)phenyl)diazenyl)naphthalen-1-yl 4-methoxybenzoate (**I_6/a_**) for the heating and cooling cycles. The N–mesophase schlieren textures observed under POM for the analogues **I_16/c_** are shown in Figure 3. The mesophase transition temperatures and their associated enthalpies were determined by DSC and are presented in Table 1. All designed derivatives, bearing the central naphthyl moiety, exhibited two transition heating and cooling peaks on DSC thermogram scans. These were ascribed to a Cr-to-N mesophase and an N-to-isotropic liquid transition upon heating and during cooling, which were reversed on cooling from isotropic liquid –N and N–Cr phases. The DSC results agreement with the mesophase textures identified by POM measurements. The transition temperatures derived from the DSC measurements of synthesized compounds are graphically represented in Figure 4. Table 1 and Figure 4 reveal that all the prepared laterally substituted compounds (**I_n/x_**) possessed a purely wide enantiotropic N mesophase. The derivative with the shortest terminal chain (**I_6/a_**), having an electron donating group, displayed the highest thermal N stability at 199.6 °C and the broadest nematic range at 70.4 °C. In contrast, the longer analogue, **I_16/a_**, possessed the lowest nematic stability and range at 119.7 and 32.7 °C, respectively. For an electron-withdrawing group, the compound **I_6/b_** (X = F) showed a nematogenic range at 40.0 °C and N stability near 154.1 °C. Meanwhile, the longest terminal chain length analogue (**I_16/c_**, X = Cl) exhibited nematic thermal stability near 158.4 °C with a N mesophase range at 51.3 °C. The mesomorphic stability of the materials was enhanced by an incremental rise in the polarity or polarizability of the mesogenic core of the compound. Thus, the electron-donating (CH_3_O) and electron-withdrawing (F and Cl) groups influenced the mesophase thermal stability and displayed a purely nematic mesophase. Moreover, it was documented in [43,58] that the stability of a formed mesophase and its type are mainly dependent upon the molecular dipole moment of the mesogenic core of the compound. This, in turn, depends on the attached terminal polar substituent and the steric one, which vary according to the location and size of the group. Thus, the insertion of a terminal polar substituent having a different volume and polarity into an azo/ester system that has terminal alkoxy chains of proportionate length, affected the mesophase transition phenomena, which depended on the location and mesomeric function of the selected substituent (i.e., donating (CH_3_O) or withdrawing (F and Cl moieties). Furthermore, the introduction of terminals into an LC material will have two opposing effects: first, it will decrease the phase stability due to the steric effect of the terminal substituent [59,60,61,62,63]; secondly, the molecular anisotropy will increase or decrease depending on the polarizing effect of the substituent. The dipole moment of the whole compound depends mainly on the position and polarity of the attached lateral and terminal groups. In addition, the terminal and lateral moieties played an essential role in the observed melting temperatures of the synthesized derivatives. Table 1 and Figure 4 show the decrease in the nematic range and stability of the laterally substituted derivatives in the following order: **I_6/a_** > **I_16/c_** > **I_6/b_** > **I_16/a_**.

Table 1 also shows the normalized entropy changes, ΔS/R, in the investigated derivatives (**I_n/x_**). The data revealed that the N–I transition entropy changes showed relatively lower values. The estimated entropy changes values are independent on alkoxy chain length [64,65]. Additionally, these results were agreement with previous reports [66,67,68]. The stereo configurations of the central naphthalene moiety and terminal polar groups played an important role in the predicted thermal parameters, which will be discussed in the computational section. Moreover, the thermal cis/trans isomerization of the azo group was an essential factor in the lower entropy changes observed, as documented in previous studies [65,69,70,71]. Moreover, due to their nematic nature, of the mesophase, they exhibited the lowest order mesophase.

### 3.2. Comparison of the Investigated Derivatives (I_n/x_) with Previously Prepared Series

**Scheme 3:**
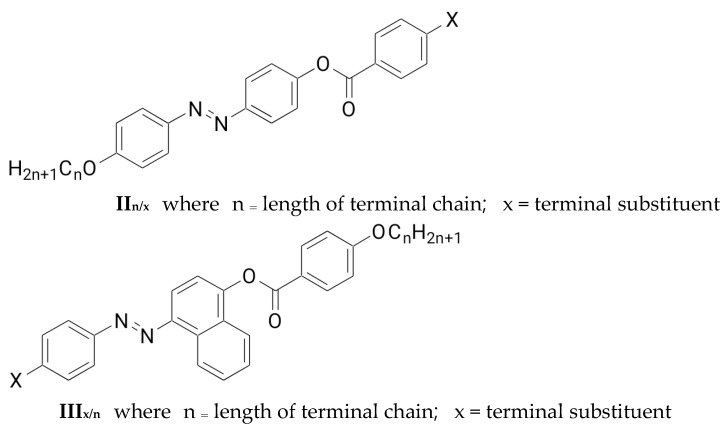
Molecular structures of series **II_n/x_** and **III_x/n._**

The location of lateral substitution strongly affects the mesomorphic behavior of LC materials. To evaluate the effect of the extra fused ring on the mesomorphic behavior on the mesogenic core of the molecule, an initial comparison is made between the derivatives (**I**_n/x_) and their corresponding laterally neat homologues bearing the central benzene ring (**II**_n/x_, Scheme 3) [58]. The homologues series **II**_n/x_ exhibiting dimorphic phases (SmA and N) depending on the length of terminal chain (n). The comparison indicated that the incorporation of the extra fused ring in the central core disrupted smectic A molecular packing and gave only resulted in the N mesophase. A second comparison is then made between the present compounds (**I**_n/x_) and their corresponding isomers bearing the central benzene ring (**III**_x/n_, Scheme 3) [57]. The homologues **III**_x/n_ series were observed to have nematognic phases with higher thermal stabilities than the present investigated series **I**_n/x_. The results revealed that the positional exchange of azo/ester linkages decreased the thermal stability of the formed mesophases.

### 3.3. Geometrical Structures and DFT Investigations

**Figure 5:**
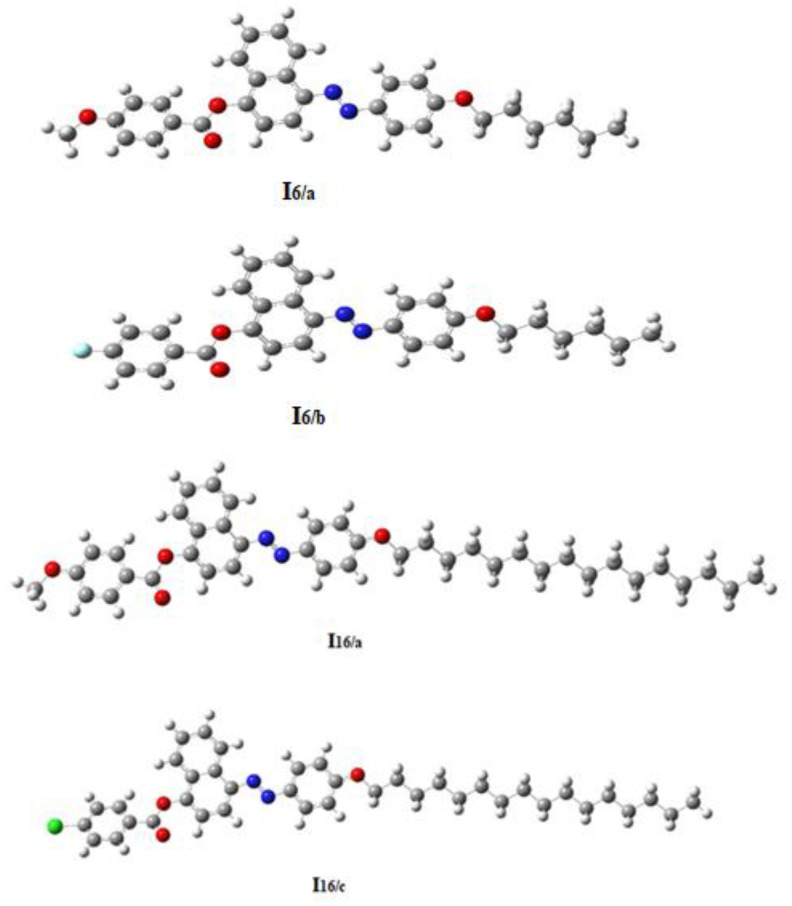
Optimum structures for molecules of the derivatives **I_n/x_**, calculated at a B3LYP/6–311G** level.

Computational calculations were carried out for all synthesized central naphthyl derivatives (**I_n/x_**) via the DFT method to correlate the predicted quantum chemical parameters and the experimental data. The DFT calculations were performed in the gas phase with a DFT/B3LYP program at a 6–311G** basis set. The prepared materials (**I_n/x_**) displayed a mesomorphic behavior, this confirming their existence in a planar conformation. Figure 5 represents the optimized geometrical structure of each compound confirmed to be stable by frequency calculation; no imaginary frequency was predicted for any member. The zero-point energy and other calculated quantum thermal parameters are summarized in Table 2 and Table 3. It can be seen from Figure 5 that all designed compounds were linear and planar. Additionally, the length of the terminal alkoxy chains had no significant effect on the planarity of the aromatic rings. It was found that, in [72], the planarity of the mesogenic cores planarity of the LC compounds was influenced by the mesomeric nature of the attached polar group. Hence, the conjugated π–cloud interactions resulting from the terminal polar substituents offered high thermal N stability and range with suitable geometrical parameters in the investigated derivatives. On the other hand, the zero-point energy and other calculated thermodynamic parameters listed in Table 2 depend on the nature of terminal substituent X. They were predicted to increase with the increasing the electron-donating mesomeric nature.

As can be seen in Table 3, the calculated ionization potentials (IE) for the terminal methoxy compounds (**I_6/a_** and **I_16/a_**)_)_ have lower values, which indicate that they are more basic than other electron-withdrawing derivatives [73]. Moreover, the predicted polarizability listed in Table 3 decreased in the order of **I_16/a_** > **I_16/c_** > **I_6/a_** > **I_6/b_**, showing that the changing values may be attributed to the aspect ratio of each molecule. As the molecular structure aspect ratio increased, the space filling of the mesomorphic compound also increased, resulting in enhanced polarizability. In general, lateral and terminal substituent polarity, polarizability, rigidity, and the shape of the liquid crystalline molecules are essential parameters for forming mesophases of specific types and thermal stabilities. The results in Table 3 also showed that the longer terminal chain derivatives (**I_16/a_** and **I_16/c_**) had lower values of predicted total energy (−1963.82 and −2308.95 Hartree). Thus, the e terminal aggregation strength increased along with the length of the alkoxy chain, but with a decrease in total thermodynamic energy. It was reported that [64,65], the terminals of the chains always change their conformation dynamically and are randomly pointed out within the chain-layer. The random packing of the chains results in the loss of the two-dimensional symmetry within the chain-layer.

## 4. Conclusions

Four new mesomorphic derivatives based on an extra laterally fused ring in the central of a molecule, namely 4-((4-(alkoxy)phenyl)diazenyl)naphthalen-1-yl 4-substitutedbenzoate (**I_n/x_**), were synthesized and mesomorphically investigated, as well as theoretically evaluated. Molecular structure elucidation was carried out by elemental analyses and FTIR and NMR spectroscopy. Mesomorphic examinations of the prepared compounds were measured via DSC and POM. Evaluations of the DSC and POM investigations revealed that all the laterally substituted derivatives synthesized were enantiotropic, exhibiting a purely nematic mesophase with high thermal stability and a broad range. The size and mesomeric effects of different terminal polar groups (CH_3_O, F, Cl) participated in the stabilization of the molecule, which achieved higher thermal N stability than the corresponding previously reported molecules. Computational DFT calculations indicated that the rigidity of the molecule increased after the extra fused ring was attached in the center of the structural shape, thus affecting the thermal and geometrical parameters. Moreover, the predicted energy gaps confirmed that the **I_16/c_** derivative was more reactive than the other compounds.

## Figures and Tables

**Table 1:** Mesomorphic transition temperatures (T, °C), enthalpy (**ΔH**, kJ/mole) of transition, normalized entropy(**ΔS**_Nematic–Iso_**/R**) of transition, and N temperature range, ΔT_,_ for compounds **I_n/x_**.

Compound.	X	T_Crystal–Nematic_	ΔH_Crystal–Nematic_	T_Nematic–Iso_	ΔH_Nematic–Iso_	ΔS_Nematic–Iso_/R	ΔT
I_6/a_	–OCH_3_	129.2	45.78	199.6	1.93	0.49	70.4
I_6/b_	–F	114.1	39.35	154.1	1.19	0.34	40.0
I_16/a_	–OCH_3_	87.00	36.38	119.7	2.36	0.72	32.7
I_16/c_	–Cl	107.1	36.96	158.4	2.30	0.64	51.3

Iso denotes isotropic liquid phase.

**Table 2:** Thermal parameters at 25 °C calculated using the B3LYP/6–311G ** method.

Comp.	ZPE (Kcal/Mol)	Thermal Energy (Kcal/Mol)	Enthalpy (Kcal/Mol)	Gibbs Free Energy (Kcal/Mol)	Entropy (Cal Mol/k)
I_6/a_	339.142	359.971	360.563	294.367	222.023
I_6/b_	313.606	333.312	333.905	270.090	214.037
I_16/a_	518.170	547.563	548.155	460.300	294.671
I_16/c_	491.790	520.310	520.903	434.582	289.519

**Table 3:** Total energy (H), E_HOMO_ (ev), E_LUMO_ (ev), ∆E (ev), dipole moment (D), ionization energy (ev), electron affinity (ev), and polarizability (Bohr^3^) calculated using B3LYP/6–311G** method for the series **I_n/x_** homologues.

Comp.	Total Energy (Hartree)	E_HOMO_ (ev)	E_luMO_ (ev)	∆E (ev)	Dipole Moment (Debye)	IE (ev)	EA (ev)	Polarizability Bohr^3^
I_6/a_	−1570.995	–5.632	–2.439	3.193	1.7620	5.632	2.439	451.01
I_6/b_	−1555.767	−5.774	−2.582	3.192	3.3197	5.774	2.582	424.69
I_16/a_	−1963.817	–5.627	–2.439	3.188	1.6794	5.627	2.439	571.88
I_16/c_	−2308.947	−5.791	−2.614	3.177	3.7771	5.791	2.614	561.58

Abbreviations: E_HOMO_ denotes to the energy of the highest occupied molecular orbital, E_LUMO_ denotes to the energy of the lowest unoccupied molecular orbital and ∆E = E_LUMO_–E_HOMO_ the orbital energy gap.

## Data Availability

The data presented in this study are available on request from the corresponding author.

## Supplementary Materials

The following are available online at https://www.mdpi.com/article/10.3390/ma14102587/s1. The synthetic and characterization details of investigated compounds. **1**. Materials; **2**. Synthesis of 4-((4-alkoxyphenyl)diazenyl)naphthalen-1-ol; **3**. Synthesis of 4-((4-(alkoxy)phenyl)diazenyl)naphthalen-1-yl 4-substitutedbenzoate In/x; **4**. Characterization; **5**. Computational Method.

## Sample Availability

Samples of the **I_n/x_** compounds are available from the authors.
