# Supplementary material for: Synthetic, Mesomorphic, and DFT Investigations of New Nematogenic Polar Naphthyl Benzoate Ester Derivatives
Source: Materials (Basel). 2021 May 16;14(10):2587. doi: 10.3390/ma14102587 (PMC8156059; doi:10.3390/ma14102587)
Supplement: Supplementary file 1 [file materials-14-02587-s001.zip › materials-1207179-supplementary.pdf]

Supplementary Data

Synthetic, Mesomorphic and DFT Investigations of New Nematogenic Polar Naphthyl Benzoate Ester Derivatives

Salma A. Al-Zahrani [4], Hoda A. Ahmed [2,3*], Mohamed A. El-atawy [3,4*], Khulood A. Abu Al-Ola[5] and Alaa Z. Omar[4]

1. Department of Chemistry, College of Sciences, University of Ha'il, Ha'il 2440, Saudi Arabia, s.alzahrane@uoh.edu.sa
2. Department of Chemistry, Faculty of Science, Cairo University, Cairo 12613, Egypt, (HAA); ahoda@sci.cu.edu.eg
3. Chemistry Department, Faculty of Science, Taibah University, Yanbu, 46423, Saudi Arabia
4. Chemistry Department, Faculty of Science, Alexandria University, P.O. 426 Ibrahemia, Alexandria, 21321, Egypt, mohamed.elatawi@alexu.edu.eg
5. Department of Chemistry, College of Science, Taibah University, 30002 Al-Madinah Al-Munawarah, Saudi Arabia, (Kh.A) Kabualola@taibahu.edu.sa.

* Correspondence: H. A. Ahmed; ahoda@sci.cu.edu.eg , M. A. El-atawy; mohamed.elatawi@alexu.edu.eg,

1. Materials

4-hexyloxyaniline, 4-hexadecyloxyaniline, 1-naphthol, 4-anisic acid, 4-chlorobenzoic and 4-fluorobenzoic acid were purchased from Sigma Aldrich (Germany). N,N'- dicyclohexylcarbodiimide (DCCD), 4-dimethylaminopyridine (DMAP), dichloromethane and ethanol were purchased from Aldrich (Wisconsin, USA).

2. Synthesis of 4-((4-alkoxyphenyl)diazenyl)naphthalen-1-ol

A solution of 4-alkoxyaniline (10.0 mmol) in hydrochloric acid was cooled in an ice-salt bath at 0-5°C. The amine solution was then added to a cold solution of sodium nitrite (10.2 mmol) at 0-5° C. The resulted mixture was stirred for 0.5h and the temperature was kept below 5°C. The formed diazonium salt solution was then added dropwise to a aqueous solution of 1-naphthol/ NaOH (10.0 mmol/ 10 mmol). The resulted solution was vigorously stirred at 0-5 °C for 2 h, while the pH of the reaction mixture was maintained at 7-8 by simultaneous addition of potassium carbonate solution (0.5 M). The progress of the reaction was monitored by TLC and then a crude dye was filtered, washed with hot water for several times then recrystallized from water/ethanol mixture.

3. Synthesis of 4-((4-(alkoxy)phenyl)diazenyl)naphthalen-1-yl 4-substitutedbenzoate In/x

Molar equivalents of 4-((4-alkoxyphenyl)diazenyl)naphthalen-1-ol and the corresponding aromatic carboxylic acid namely, 4-anisic acid, 4-cholrobenzoic acid or 4-fluorobenzoic acid (0.01 mole) were dissolved in 25 ml dry methylene chloride. N, N′-dicyclohexylcarbodiimide (DCCD) (0.02 mol) and few crystals of 4-dimethylaminopyridine (DMAP), as catalyst, were added. The solution left to stand for 72 hours at room temperature with continuous stirring. The byproduct, dicyclohexylurea (DCU), separated was then filtered off and the filtrate was then evaporated. The obtained solid residue was recrystallized twice from ethanol to give TLC pure products.

4. Characterization

Melting points were determined by MEL-TEMP II melting point apparatus in open glass capillaries and were uncorrected. The IR spectra were recorded as potassium bromide (KBr) discs on a Perkin-Elemer FT-IR (Fourier-Transform Infrared Spectroscopy), college of Science, Taibah University. The NMR spectra were carried out at ambient temperature (~25 °C) on a (JEOL) 500 MHz spectrophotometer using tetramethylsilane (TMS) as an internal standard, NMR Unit, Faculty of Science, King Abdul-Aziz University. Chemical shift was recorded as δ values in parts per million (ppm), and the signals were reported as s (singlet), d (doublet), t (triplet) and m (multiplet). Elemental analyses were analyzed at the Micro analytical Unit, Faculty of Science, Cairo University.

TA Instruments Co. (Q20 Differential Scanning Calorimeter, DSC; USA) was used for recording phase transitions. DSC calibration was carried using lead and indium melting temperature and enthalpy. Samples of 2–3 mg were used in aluminum pans for DSC investigation. The heating rate was 10°C/min in nitrogen gas as an inert atmosphere (30 ml/min). All transitions measured for the second heating scan.

Transition temperatures for the prepared compounds were checked and phases identified by Polarized optical microscope (POM, Wild, Germany) attached with Mettler FP82HT hot stage.

5. Computational Method

All DFT calculations were performed using the Gaussian 09 software. A conformational search for obtaining the most stable conformer was done using the semi-empirical PM3 method. The most stable conformer was subjected to full geometrical optimizations using the DFT and Becke's three-parameter hybrid exchange functional in combination with the gradient-corrected correlation functional of Lee, Yang and Parr B3LYP/6-311G** method without any constraints to calculations. All calculated structures were found to be true minima, i.e., no imaginary frequencies were observed after the ground state geometry optimization.
